# Finding the right BCR-ABL1 tyrosine kinase inhibitor: a case report of successful treatment of a patient with chronic myeloid leukemia and a V299L mutation using nilotinib

**DOI:** 10.1186/s12885-018-5004-3

**Published:** 2018-11-12

**Authors:** Radowan Elnair, Ahmed Galal

**Affiliations:** 1 Department of Internal Medicine, Sanford School of Medicine, University of South Dakota, Sioux Falls, SD USA; 2 Division of Hematologic Malignancies and Cellular Therapy, Department of Medicine, Duke University School of Medicine, Durham, NC USA

**Keywords:** Chronic myeloid leukemia, Nilotinib, Drug-resistant BCR-ABL mutations, V299L, Dasatinib

## Abstract

**Background:**

Chronic myeloid leukemia can be effectively treated with BCR-ABL1 tyrosine kinase inhibitors. However, BCR-ABL1 mutations can develop and cause secondary resistance to these inhibitors. For each of the available BCR-ABL1 inhibitors, certain mutations are known to be associated with resistance, although most mutations that confer resistance to one tyrosine kinase inhibitor remain sensitive to one or more of the other available inhibitors. For patients displaying poor response or loss of response to frontline treatment, the possibility that they have developed a new BCR-ABL1 mutation must be considered, and selection of a second-line treatment must consider the patient’s mutational profile. Here we describe a case in which a patient developed a V299L mutation; although this mutation is known to be associated with resistance to dasatinib while remaining sensitive to nilotinib, limited information is currently available regarding the use of second-line nilotinib following development of a V299L mutation while receiving dasatinib.

**Case presentation:**

A 73-year-old man presenting with fatigue and drenching night sweats lasting for 2 weeks was diagnosed with chronic myeloid leukemia based on an analysis of a bone marrow biopsy and detection of the *BCR-ABL1* fusion gene in peripheral blood. The patient initiated frontline treatment with dasatinib. A good treatment response was seen initially, with a complete hematologic response by month 2 of treatment. By month 20 however, *BCR-ABL1* transcript levels rose markedly, and a mutational analysis revealed a BCR-ABL1 V299L mutation. Based on the identification of this specific mutation, the patient switched treatment to nilotinib; by month 18 of nilotinib treatment, the patient achieved a deeper reduction in *BCR-ABL1* transcript levels than was seen with dasatinib. To date, in month 34 of treatment with nilotinib, the patient has shown good tolerance of the drug and has no clinical evidence of disease progression.

**Conclusions:**

Our case report illustrates the benefit of having multiple drugs available to treat chronic myeloid leukemia, each with the ability to inhibit a distinct set of BCR-ABL1 mutations. This patient’s case suggests that switching to nilotinib can be an effective treatment option for patients who develop a BCR-ABL1 V299L mutation while receiving dasatinib.

## Background

Chronic myeloid leukemia (CML) is characterized by the presence of the Philadelphia chromosome, which is generated by a reciprocal translocation between chromosomes 9 and 22: t(9;22)(q34;q11). This translocation produces the *BCR-ABL1* fusion gene, which encodes the constitutively active BCR-ABL1 tyrosine kinase [1, 2]. Currently, 5 BCR-ABL1 tyrosine kinase inhibitors (TKIs) are available to treat patients with CML. Imatinib was the first TKI developed and was approved for frontline use after demonstrating remarkably improved efficacy over all previous standards of care [3]. The second-generation TKIs nilotinib, dasatinib, and bosutinib were approved for frontline use after demonstrating improved efficacy over imatinib in randomized clinical trials [4–6]. Ponatinib, a third-generation TKI, is available to treat patients with CML in later-line settings [7]. Due to the success of these TKIs, patients with CML now have life expectancies comparable to those in the general population [8].

Responses to TKI therapy are typically monitored using real-time quantitative polymerase chain reaction (RQ-PCR) methods to quantify the number of *BCR-ABL1* transcripts in peripheral blood; RQ-PCR results are then converted to the standardized International Scale (IS) to evaluate the level of response to treatment [7]. For example, a *BCR-ABL1* level of 0.1% on the IS indicates that a patient’s *BCR-ABL1* transcript level is 0.1% of that in the reference sample representing a standardized baseline, pretreatment level [7, 9]. The National Comprehensive Cancer Network (NCCN) provides guidelines for determining whether a patient is responding appropriately to treatment based on his or her *BCR-ABL1* levels at designated time points [7]. Currently, the NCCN recommends a change in treatment for patients with *BCR-ABL1* transcript levels > 10% on the IS after 6 months of treatment or > 1% after > 15 months; the NCCN also notes that a switch may be appropriate for patients with *BCR-ABL1* levels > 10% on the IS after 3 months or > 1% after 12 months [7]. Furthermore, for patients meeting any of these criteria, the NCCN recommends evaluation of treatment adherence and potential drug interactions, as well as a BCR-ABL1 mutational analysis [7].

Development of point mutations in BCR-ABL1 is a frequent cause of secondary drug resistance in CML and is associated with poor prognosis and disease progression [7, 10–15]. When a BCR-ABL1 mutation is detected in a patient with CML, a change in therapy to a different TKI is recommended [7, 14]. Because each BCR-ABL1 TKI is active against a distinct set of BCR-ABL1 mutants, a patient who develops a mutation that confers resistance to their frontline TKI can often be switched to a second-line TKI that will provide continued disease control [7, 14]. For example, the V299L mutation confers resistance to dasatinib [14, 16] and bosutinib [17], but it is not associated with resistance to nilotinib, and high rates of response to nilotinib have been observed in patients with V299L mutations [17, 18].

We describe a patient newly diagnosed with CML in chronic phase who initiated treatment with frontline dasatinib and switched to nilotinib following the development of secondary resistance and the identification of a V299L mutation. This case report adds to the relatively small body of knowledge regarding outcomes in patients who have switched from dasatinib to nilotinib following the identification of a V299L mutation.

## Case presentation

A 73-year-old white male patient was referred to the hematology clinic due to a significantly elevated white blood cell (WBC) count that was detected following presentation with fatigue and drenching night sweats lasting 2 weeks. Night sweats and fatigue can be signs of an infection, malignancy, or hormonal abnormality, or they can be side effects of medication. For patients presenting with these symptoms, likely potential diagnoses include tuberculosis, HIV, abscesses, infective endocarditis, lymphoma or leukemia, hyperthyroidism, pheochromocytoma, or carcinoid syndrome.

The patient’s medical, surgical, social, and family histories are reported in Table 1. There were no relevant past interventions. To further evaluate and diagnose the patient’s condition, we performed a complete blood count (CBC; Table 1) and peripheral blood smear. The peripheral blood smear showed a number of teardrop cells. Following the CBC and peripheral blood smear results, an abdominal ultrasound was performed and showed splenomegaly of approximately 16 cm. The lactate dehydrogenase level was also examined and found to be elevated at 1005 U/L.

**Table 1:** Patient’s histories and clinical features at presentation

Patient histories	
Medical history	Chronic obstructive pulmonary disease and surgically treated prostate cancer
Surgical history	Prostatectomy, cholecystectomy, and hernia repair
Social history	88 pack-years of smoking and consumption of 2 alcoholic beverages per day; no history of illicit drug use
Family history	No family history of blood disorders, clotting disorders, or malignancies
Clinical features at presentation	
White blood cell count, cells/μL	147,000
Basophils, %	10
Absolute neutrophil count, cells/μL	116,210
Platelet count, platelets/μL	230,000
Hemoglobin, g/dL	13.1
Abdominal ultrasound	Splenomegaly of ≈ 16 cm
Lactate dehydrogenase, U/L	1005

The patient’s clinical presentation, elevated WBC count, splenomegaly, and peripheral blood smear results were suggestive of a myeloproliferative disorder, with CML suggested based on the peripheral blood smear and cytological analyses. To confirm a diagnosis of CML, a bone marrow biopsy and PCR test on peripheral blood for the *BCR-ABL1* fusion gene were conducted. Examination of cells from the bone marrow biopsy showed hypercellular marrow, with increased megakaryocytes, increased and left-shifted granulopoiesis, markedly decreased erythropoiesis, eosinophilia, decreased iron, severe reticulin fibrosis, and approximately 5% blasts. A CD34 immunohistochemical stain showed scattered CD34-positive blasts comprising approximately 5% of the overall marrow cellularity, with variable distribution of blasts without clusters. A cytogenetic analysis could not be performed owing to a culture failure, likely resulting from a clotted specimen. However, a PCR test was positive for the *BCR-ABL1* fusion gene.

The patient was in chronic phase of CML and according to his Sokal risk score, was classified as low risk. The Kaplan-Meier-estimated 5-year overall survival rate for patients in his age group (65–74 years old) diagnosed with CML in 2000 (before the introduction of TKIs) compared with those diagnosed with CML in 2005 (after the introduction of TKIs) was reported as 38.1% versus 51.2%, respectively (hazard ratio for mortality, 0.692; 95% CI, 0.518–0.924; *P* = .0126) [19]. Available treatments and their side effect profiles were discussed with the patient, and he elected to proceed with dasatinib treatment.

The patient was started on dasatinib 100 mg once daily. Treatment adherence and tolerability were reviewed during each of his follow-up visits to the clinic; the number of pills remaining, if any, was always verified with the patient. He tolerated the treatment well and within 2 months experienced a complete hematologic response. The patient’s response was monitored by evaluating *BCR-ABL1* transcript levels; isolated RNA was reverse transcribed, after which the complementary DNA was amplified by RQ-PCR for the major and minor *BCR-ABL1* fusion genes. The patient had no evidence of disease progression and achieved a molecular response of *BCR-ABL1* < 10% on the IS during month 5 of treatment. For patients with this level of response, the NCCN recommends continuing the current treatment, with ongoing monitoring of response levels [7]. By approximately month 8 of treatment, *BCR-ABL1* levels increased slightly from 2.40 to 3.59% on the IS; however, a subsequent assessment 4 weeks later showed a reduction of *BCR-ABL1* levels to 2.99% on the IS.

**Fig. 1:**
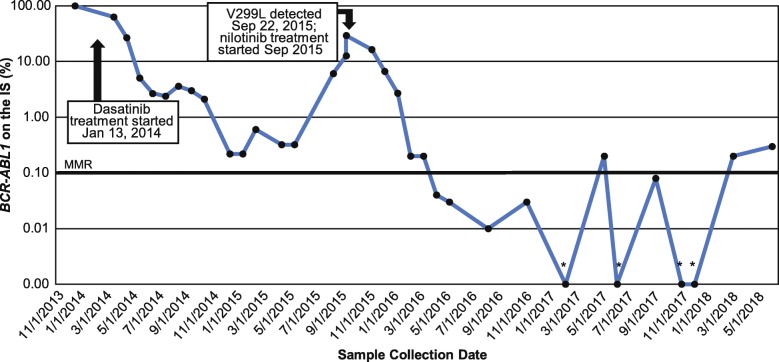
*BCR-ABL1* levels over time. IS, International Scale; MMR, major molecular response (*BCR-ABL1* ≤ 0.1% on the IS). * *BCR-ABL1* = 0.00% on the IS

Increasing *BCR-ABL1* levels can be an early sign of treatment resistance [20]. In prior studies, a ≥ 2-fold increase in *BCR-ABL1* levels in single or serial samples was shown to be predictive of BCR-ABL1 mutations [20, 21], which are a frequent cause of TKI resistance [7, 10–15]. The NCCN recommends additional testing in patients with a 1-log increase in *BCR-ABL1* levels and loss of MMR to determine if a change in treatment is needed [7]. However, in this case, the patient’s increasing *BCR-ABL1* levels at month 8 of treatment were below the 2-fold and 1-log thresholds, and they spontaneously improved by the subsequent assessment. At month 12 of treatment, a bone marrow biopsy revealed no increase in blasts (< 1%) and adequate erythropoiesis and granulopoiesis, while RQ-PCR showed a *BCR-ABL1* level of 0.22% on the IS, which is close to a major molecular response (*BCR-ABL1* ≤ 0.1% on the IS). The favorable results of the bone marrow biopsy and the RQ-PCR results indicated that the patient was responding well to treatment. The patient continued treatment with dasatinib (Fig. 1).

At month 20 of dasatinib therapy, another increase in *BCR-ABL1* levels was detected (from 0.32% on the IS at month 16 to 6.09% at month 20). However, the patient showed no clinical evidence of disease progression, remained on treatment with good adherence, and had normal CBC levels. He was therefore kept on dasatinib treatment, and his *BCR-ABL1* levels were assessed again at month 21. This assessment showed that his *BCR-ABL1* levels had increased further, to 12.77% on the IS. A bone marrow biopsy revealed no evidence of acute leukemia. Cytogenetic analysis showed that 10 of 20 cells were positive for the Philadelphia chromosome; 10 normal cells were observed. Unlike the earlier increase in *BCR-ABL1* levels, this increase was substantial enough to trigger BCR-ABL1 mutational analysis despite the absence of clinical evidence of disease progression. Genetic sequencing of a bone marrow aspirate sample detected a V299L mutation in the BCR-ABL1 kinase domain. Low levels of an insertion event, during which 35 nucleotides from *ABL1* intron 8 were inserted at the normal exon 8 to exon 9 splice junction, were also detected; the clinical significance of this is unknown. The NCCN recommends switching patients with V299L mutations to nilotinib [7]. In accordance with these treatment guidelines, the patient was switched to nilotinib 400 mg twice daily.

After starting nilotinib 400 mg twice daily, the patient developed abdominal pain, slightly elevated amylase and lipase levels, and profound fatigue. Due to these adverse events, the nilotinib dose was temporarily reduced to 200 mg twice daily and then escalated to a 300-mg twice-daily maintenance dose. RQ-PCR testing at month 18 revealed a *BCR-ABL1* level of 0.00% on the IS, a greater reduction than was previously achieved with dasatinib. To date, the patient has remained on nilotinib 300 mg twice daily and has demonstrated good tolerability of the drug, no recurrence of abdominal pain or fatigue, and no clinical evidence of disease progression. *BCR-ABL1* levels rose to 0.20% on the IS at month 21 of nilotinib but returned to 0.00% on the IS the following month. In the latest assessment, at month 34 of treatment, the patient had *BCR-ABL1* levels of 0.30% on the IS, up from 0.00% on the IS at month 28. He showed no evidence of cytogenetic or hematologic relapse and is being periodically followed at the clinic per the NCCN guidelines [7].

## Discussion and conclusions

Today, most patients with CML have good long-term prognoses, including a life expectancy comparable to that of the general population [8]. However, regular monitoring of these patients is important to enable a timely response to any signs of resistance to treatment or disease progression, such as increasing *BCR-ABL1* levels [7, 22]. Because BCR-ABL1 mutations are frequently present in patients who develop TKI resistance, mutational analysis is recommended for patients with loss of response [7, 22]. The presence of BCR-ABL1 mutations can indicate that a patient is at an increased risk of progression to advanced phases of CML [22], which lead to a substantial reduction in survival duration [23]. Thus, treatment switch to a TKI that is effective against the specific mutation detected is crucial.

Although nilotinib is recommended for patients with V299L mutations, limited data are available on outcomes in patients who switch from dasatinib to nilotinib due to this mutation. Several cases of patients with V299L mutations responding to nilotinib following a switch from dasatinib have been reported [17, 24]. The patient reported in this case report was not able to tolerate the target dose of nilotinib (400 mg twice daily) due to side effects. However, he had good tolerability of and a good response to the reduced dosage of 300 mg twice daily. While this individual case report has the limitation of lacking statistical power, it concurs with the current recommendations for consideration of use of nilotinib in patients with a V299L mutation [7]. The remarkable response to nilotinib observed in our patient illustrates the benefits of having several therapeutic options available to effectively treat CML in chronic phase and the importance of considering each patient’s mutational status and medical history.

## Abbreviations

CBC: Complete blood count
CML: Chronic myeloid leukemia
IS: International scale
NCCN: National Comprehensive Cancer Network
RQ-PCR: Real-time quantitative polymerase chain reaction
TKI: Tyrosine kinase inhibitor
WBC: White blood cell
